# Advances in the Determination of Anabolic-Androgenic Steroids: From Standard Practices to Tailor-Designed Multidisciplinary Approaches

**DOI:** 10.3390/s22010004

**Published:** 2021-12-21

**Authors:** Lukáš Huml, Jan Tauchen, Silvie Rimpelová, Barbora Holubová, Oldřich Lapčík, Michal Jurášek

**Affiliations:** 1 Department of Chemistry of Natural Compounds, Faculty of Food and Biochemical Technology, University of Chemistry and Technology Prague, 16628 Prague, Czech Republic; oldrich.lapcik@vscht.cz (O.L.); michal.jurasek@vscht.cz (M.J.); 2 Department of Food Science, Faculty of Agrobiology, Food and Natural Resources, Czech University of Life Sciences Prague, 16500 Prague, Czech Republic; tauchen@af.czu.cz; 3 Department of Biochemistry and Microbiology, Faculty of Food and Biochemical Technology, University of Chemistry and Technology Prague, 16628 Prague, Czech Republic; barbora.holubova@vscht.cz

**Keywords:** anabolic-androgenic steroids, biosensors, chemically designed sensors, antibodies, specific detection, fluorescent sensors, immunoassays, chromatographic detection, immunosensors, oligonucleotide-based approach

## Abstract

Anabolic-androgenic steroids (AASs), a group of compounds frequently misused by athletes and, unfortunately, also by the general population, have lately attracted global attention; thus, significant demands for more precise, facile, and rapid AAS detection have arisen. The standard methods ordinarily used for AAS determination include liquid and gas chromatography coupled with mass spectrometry. However, good knowledge of steroid metabolism, pretreatment of samples (such as derivatization), and well-trained operators of the instruments are required, making this procedure expensive, complicated, and not routinely applicable. In the drive to meet current AAS detection demands, the scientific focus has shifted to developing novel, tailor-made approaches leading to time- and cost-effective, routine, and field-portable methods for AAS determination in various matrices, such as biological fluids, food supplements, meat, water, or other environmental components. Therefore, herein, we present a comprehensive review article covering recent advances in AAS determination, with a strong emphasis on the increasingly important role of chemically designed artificial sensors, biosensors, and antibody- and fluorescence-based methods.

## 1. Introduction

Accurate, simple, and rapid determination of AASs is increasingly coming to the forefront of scientific and social interests, not only because this group of substances is abused by athletes to gain a competitive advantage, but also because their abuse is established in the general population [1]. Due to the potential of AASs to increase proteosynthesis in the skeletal muscle and, thus, overall strength [2], AASs are largely sought after by young boys, adult men, and women [3,4,5]. These individuals are usually united by a desire for an ideal figure, self-confident behavior, and better social status. However, they often ignore the possible adverse effects and the severity of their actions, and even if they know about them, they are willing to take risks. These include possible behavioral changes, anxiety [6], increased aggression [7], hepatotoxicity [8], cardiotoxicity [9], abnormalities of the reproductive system [10,11], and many others [12]. Particularly alarming, however, is a tendency to engage in criminal activities [13], along with the growing number of case reports of fatal medical conditions connected to the misuse of AASs [14,15,16,17,18], potentially resulting in sudden deaths [19,20,21,22]. It is also worth mentioning that, on the other hand, AASs have therapeutic potential, and cannot be easily replaced by other drugs in a range of conditions such as wasting syndromes, severe burns, muscle and bone injuries, anemia, and hereditary angioedema [23].

While the severity of the struggle against AASs at the level of sports professionals has resonated with society, and the moral aspects of doping seem to have not been underestimated, the situation regarding anabolic abuse among the general population appears different. From a broad portfolio of even unofficial sources, it is clear that the thousands of cases of anabolic doping identified by the World Anti-Doping Agency (WADA) among professional athletes each year [24] are just the tip of the iceberg of substance abuse. Sport is not only about the success of an individual or team, but also about financial gain, which only strengthens the efforts to develop strategies that cover one’s tracks when doping [25]. Globally, realistic estimates of the number of anabolic steroid users are in the millions of cases [26,27,28], and the interest in them—at least on the internet—continues to grow [29].

The misuse of prohibited AASs is supported, among other things, by their effortless availability on the internet [30]. The risk of using thus-obtained preparations also lies in their dubious origin. The content of the illegally obtained preparations very often differs from what is declared [31,32]; therefore, it often transpires that users take a different substance than they think.

A stark contrast to the intentional misuse of AASs is their undeclared occurrence in dietary supplements (DSs) [33,34,35], which is a topic we have dealt with for a long time at the University of Chemistry and Technology, Prague [36,37,38,39,40,41]. Despite the apparent threat and criminal nature of undeclared enrichment of DSs with AASs, this phenomenon has been detected worldwide from time to time during research [42], random inspections of overseeing authorities [43,44], or as a result of revealing the cause of health problems [45]. Whether the presence of AASs is caused intentionally, or by undesired contamination during production, the use of such DSs can have serious health consequences for the consumer. The inadvertent consumption of AASs not only distorts fair competition between athletes but can also have fatal consequences for their professional careers if convicted of banned doping. Due to the legislative treatment of DSs, which are not subject to mandatory testing for the presence of prohibited substances before being marketed, the question remains as to how many such harmful DSs remain undetected on the market [46].

Another critical aspect potentially affecting public health is the presence of AASs in the environment [47], drinking water [48,49], and food from animal sources [50,51]. Although in such cases AASs are usually present at very low concentrations, they may still affect the endocrine and/or reproductive systems of exposed organisms [52,53,54].

These facts represent a challenge for forensic scientists and accredited laboratories, which are utilized by anti-doping surveillance authorities and the broader scientific community to develop novel techniques for the determination of AASs, or to improve the existing methods [55]. However, in addition to advances in laboratory techniques, as with other performance-enhancing drugs, reducing the incidence and frequency of abuse will require restrictions on effortless access to AASs and, possibly, a shift in the social recognition of athletic performance and muscular appearance [56]. However, this effort will not be possible without extending the necessary techniques into our everyday lives.

For the determination of AASs, various immunoassay formats using antibody-antigen interactions have been developed over the past decades. The oldest format of an immunoassay for the determination of AASs is the radioimmunoassay (RIA), which has been used for many decades in clinical as well as in anti-doping practice due to its reliability and accuracy [57]. However, RIA is gradually being replaced by immunoassays that do not suffer from the problems associated with radioisotopes, restricting its use to specialized laboratories [58,59]. The list of immunoanalytical formats known today is rather long and has been thoroughly reviewed elsewhere [60,61]. The immunoanalytical arrangements share several valuable advantages, such as high sensitivity and time- and cost-effectiveness. However, they may also suffer from significant disadvantages in some cases, such as unsatisfactory quantification or the presence of false-positive signals due to the insufficient specificity of the antibody used. Therefore, for forensic or doping control purposes, the results obtained by these methods require further confirmation of the presence of AASs using more complex instrumental techniques, such as chromatographic methods coupled with mass detection [62].

The chromatographic separation of biological samples combined with mass detection in various configurations undoubtedly forms the basis of anti-doping control and AAS research in general. These techniques achieve high sensitivity and specificity and serve to determine synthetic and endogenous AASs. Attention in anti-doping controls is mainly paid to detecting these groups of substances and their metabolites in urine samples [63,64]. Unfortunately, even these chromatographic methods have certain limitations, which prevent their broader use; they require expensive instrumentation and highly qualified operators and are not suitable for non-target analysis. Therefore, AASs of unknown composition are not identified by these procedures [55]. An indispensable burden is also the necessity of sample preparation, which is time-consuming. Therefore, this traditional approach is not suitable for routine analysis of a large number of samples, let alone for use in fieldwork. A detailed overview of the standard methods used to determine AASs over the past decades can be found, for example, in the monographs Doping in Sports [64] or Steroid Analysis [65].

To overcome some of the aforementioned limitations and disadvantages of standard practices, researchers have developed advanced multidisciplinary approaches. The most promising of these are various types of biosensors and chemically designed artificial sensors, which show great potential to solve the problems and challenges associated with AAS determination in various matrices, without the need for complex sample processing [66]. Therefore, such multidisciplinary approaches have been increasingly coming to the forefront of interest in various applications, such as environmental monitoring, food and beverage safety, medicine, pharmacology, and forensic analysis [67].

**Figure 1:**
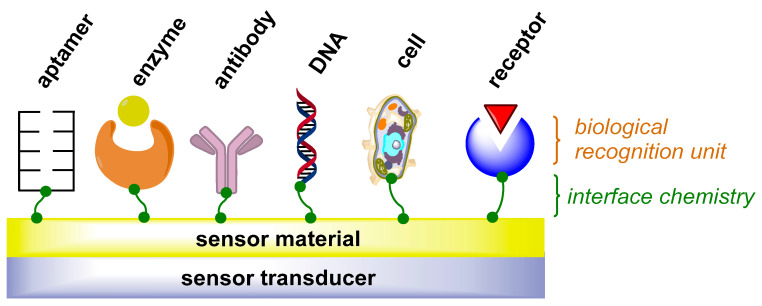
A schematic diagram of a biosensor. DNA: deoxyribonucleic acid.

This review article deals with current developments in the field of AAS determination, with the main emphasis on methods utilizing antibodies, enzymes, aptamers, oligonucleotides, cells, their receptors, and, last but not least, chemically designed artificial sensors. A schematic diagram of a biosensor is depicted in Figure 1. Since it is difficult to unambiguously classify methods for AAS determination due to the multidisciplinary character of some of them, the discussed approaches are divided mainly according to the nature of the recognition structures concerning the physical principles of the conversion of the measured quantity to the signal value.

## 2. Standard Chromatographic Methods in AAS Determination

Gas and liquid chromatography combined with mass detection in various configurations have an irreplaceable position in the determination of AASs, both in forensic and clinical practice [65]. This group of methods represents unique tools for convicting athletes of prohibited doping, and also holds an important position in the analysis of detained suspicious materials [64]. Therefore, such methods have attracted significant attention from experts worldwide. Even though AAS detection in biological fluids should be facilitated by the fact that most of them do not naturally occur in the human body, the development of these methods faces several challenges. Even more complicated is the situation with endogenous AASs, such as testosterone, which represents a particular substance in terms of determining prohibited doping. To prove the abuse of exogenous testosterone, the determination of testosterone and epitestosterone concentration ratios serves as a valid indicator. As an official method, gas chromatography/combustion/isotope ratio mass spectrometry has been introduced to distinguish between endogenous and exogenously administered testosterone [68]. The most up-to-date instrumental techniques for AAS determination regularly attract interest from several world-renowned authors; therefore, we refer to some of their works [69,70,71,72,73].

When discussing traditional chromatographic methods, thin-layer chromatography (TLC) should also be mentioned. Despite the apparent limitations of this method, TLC is one of the simplest, oldest, and most widely used separation methods, which does not require expensive equipment and, thus, is one of the most readily available analytical methods. An overview of TLC analysis of steroids, including AASs, is given in [74]. Of the current steroids, the development of a method for the densitometric determination of stanozolol is worth mentioning [75]. In this method, the limit of detection (LOD) is 1.6 ng per spot, and a good linear relationship over the range of 200–1200 ng per spot concentrations was achieved on traditional silica-gel-coated aluminum plates using petroleum ether:acetone (6:4, v/v) as the mobile phase. This method has been validated for the quantification and determination of stanozolol degradation in pharmaceutical preparations. Due to its simplicity, this method is an attractive alternative to the traditional instrumental analysis of stanozolol-containing pharmaceutical preparations. An order of magnitude higher sensitivity was achieved for testosterone in a study that used a modification of silica gel with gold nanoparticles (AuNPs), where the LOD in urine reached 0.13 ng per spot at the linear range of 1–200 ng per spot [76].

## 3. Antibody-Based Approaches for AAS Determination

Standard antibody-based methods for the determination of AASs are widely used in clinical and screening practice. For many decades, these methods have received great attention, especially for their designs which, compared to instrumental methods, enable the analysis of a larger number of samples with an order of magnitude lower cost and high sensitivity, often without the need to purify the sample. Multidisciplinary approaches in recent years have brought new procedures utilizing antibodies. In this chapter, we provide an overview of antibody-based methods, which we divide according to their setup into the following categories:

### 3.1. Immunoaffinity Columns

Immunoaffinity columns have proven their effectiveness and high specificity already in the past, which makes them among the most efficient techniques for single-step extraction of individual compounds or their classes from complex matrices [77,78]. Their advantages are simplicity and the possibility of reusing the immunosorbent. Many different methods for immobilizing antibodies or their fragments on a solid phase exist; however, they are often bound covalently [79]. Table 1 provides an overview of the few reported immunoaffinity chromatography (IAC) methods for AAS determination.

Three generations of IAC methods for the extraction of methandienone were developed by Wang et al. [80,81,82]. Their methodology included immunogen synthesis and gaining polyclonal Abs [82]. Subsequently, a transition to monoclonal Abs followed, which significantly increased the binding capacity of the immunosorbent [81], while the development of improved chitosan beads led to the homogenization and improved stability of the obtained immunosorbent [80].

IAC based on gold-coated magnetic nanoparticles for the extraction of epitestosterone from human urine yielded up to a 100-fold concentration of the target analyte in the sample prepared for HPLC analysis. Therefore, IAC based on gold-coated magnetic nanoparticles can be used to analyze samples containing epitestosterone at concentrations below the detection limit of the method [83].

### 3.2. Enzymatic Immunoassays

Undoubtedly, the most used enzyme immunoassay (EIA) design is the enzyme-linked immunosorbent assay (ELISA). In practice, several different ELISA formats have been implemented. For the detection of AASs and other low-molecular-weight substances, a format of indirect competitive ELISA is suitable. This is based on the immobilized antigen and the separation of the individual reaction steps. Characteristic features include high sensitivity and the possibility of measurement in biological or food samples of various origins [84]. In recent years, the use of chemiluminescent enzyme immunoassays (CLEIAs) in clinical diagnostics and analytical tests for food and pharmacological purposes has also become widespread; this is primarily due to their very high sensitivity, broad detection range, and, above all, the speed of their procedure, which is significantly shorter compared to conventional ELISA. Moreover, CLEIA, like ELISA, is not very demanding in terms of instrumentation [85,86]. Currently published EIAs for the determination of AASs can be found in Table 2; they differ from one another in the analyte of interest, the approach to the synthesis of immunogens and an immobilization conjugate, the origin of antibodies, the matrix for which the method can be used for a measurement, and also the specificity and sensitivity of detection.

The presented EIA methods are used for detection of the most frequently abused AASs from various matrices of animal, plant, or pharmaceutical origin, with the lowest detection limits in the order of tens of picograms per mL. Despite the efforts to overcome their most fundamental analytical limitation—i.e., the phenomenon of cross-reactivity with structurally related analytes—it appears that even the development and use of monoclonal antibodies may not lead to an absolutely specific method. On the other hand, group-specific antibodies might be useful for multianalyte detection, such as in the case of stanozolol ELISA, which also detects other orally active 17α-methylated AASs [40].

**Figure 2:**
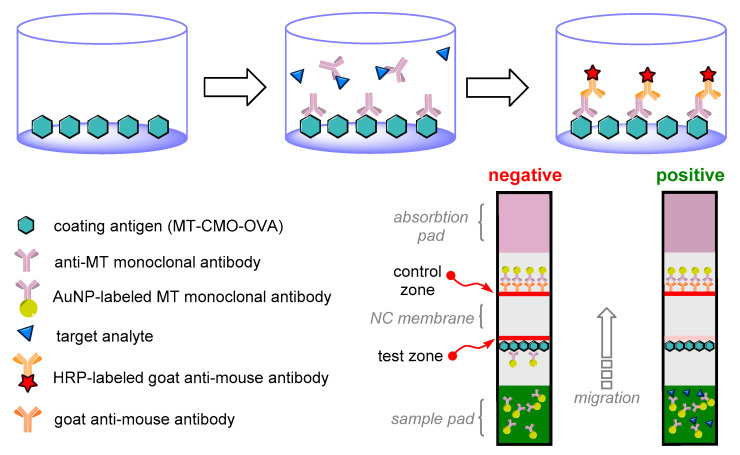
A diagram depicting the principle of indirect competitive enzyme-linked immunosorbent assay (ELISA) and the composition of an immunochromatographic test. NC: nitrocellulose; MT: methyltestosterone; MT-CMO-OVA: a conjugate of methyltestosterone-3-carboxymethyloxime with ovalbumin; mAb: mouse-derived antibody against MT; HRP: horseradish peroxidase [91].

The same work for the determination of stanozolol presents an interesting comparison provided by the antigen immobilization methodology. While using a coating with a stanozolol–protein conjugate, the ELISA achieved higher sensitivity but lower stability over time than when using a biotinylated form, for which the ELISA was less sensitive, but the parameters of the method did not change even after four months of the coated microplate’s storage [40]. The schemes of ELISA setup and measurement are given in Figure 2.

### 3.3. Lateral Flow Immunoassays

Of the available AAS immunoassay formats, the lateral flow immunoassay (LFIA, Table 3) is the simplest and the most user-friendly approach. Despite the semi-quantitative nature of this method, this strip immunoassay test enables the determination of the presence of AASs without the need for specially trained operators or requirements for any measurement equipment.

The ability to analyze liquid samples or solid sample extracts without purification is one of the undisputed advantages that LFIAs have over commonly used instrumental methods. However, immunochemical interactions are not entirely free of interferences caused by unidentified matrix compounds. Nevertheless, in LFIAs, in some cases, the movement of the sample across the membrane leads to a partial separation of the interfering compounds; therefore, the negative effect of the matrix might be less pronounced than in ELISA [89].

The presented LFIAs in Table 3 differ in the analyte of interest and the origin of the antibodies used; however, they all use gold labeling. The lowest achieved LOD for AASs that can be detected by a naked eye is 0.7 ng per mL in the case of 17α-methylated AASs such as stanozolol [41]. Compared to the currently developed ELISAs, LFIAs are generally less sensitive; on the other hand, for example, an ethanol extract of food supplements can be diluted to a lower extent for LFIA than for ELISA [89]. However, if we take into account the fact that LFIA is evaluated solely by the naked eye, and does not require any laboratory tools, it is possible to consider the detection limits of these methods as excellent. These properties may be useful for incorporating this methodology into monitoring programs—for example, to control contamination of food supplements. However, to confirm the positivity of suspect samples, the result should be verified using instrumental methods, as in the case of other Ab-based methods.

### 3.4. Immunosensors

Other immunoassay formats include immunosensors that can provide fast, cost-effective, highly sensitive, and specific assays [95]. In immunosensors, signal generation due to the complex formed between the Ab and the antigen is monitored, while among the used detection strategies belong direct, indirect, competitive, and sandwich modes [96]. In addition to traditionally used antibodies, natural single-domain nanobodies from the serum of *Camelidae* might also be employed in the detection system [97,98]. The immobilization of the Ab on the electrode surface is particularly essential during the manufacturing of this type of biosensor, affecting its performance and stability. A common tool for successful Ab immobilization on a surface is the covalent attachment of functional chemical groups such as hydroxyl, amine, or carboxyl groups on the conjugated polymers [99]. An overview of the developed immunosensors and their characteristics is given in Table 4.

Most of the immunosensors listed in Table 4 are electrochemical, most often using amperometric or electrochemical impedance spectroscopy transduction. They differ mainly in the different arrangement of the electrodes and Ab immobilization. The possibility of detecting low AAS concentrations is also given by immunosensors with optical detection based on the phenomenon of surface plasmon resonance (SPR), which is also label-free. In general, the goal of developing methods designed in this way is rapid and facile analysis without the need for sample preparation. These methodologies also share the ability to analyze small sample volumes with high sensitivity, reaching tens—in exceptional cases up to units—of picograms per mL.

**Figure 3:**
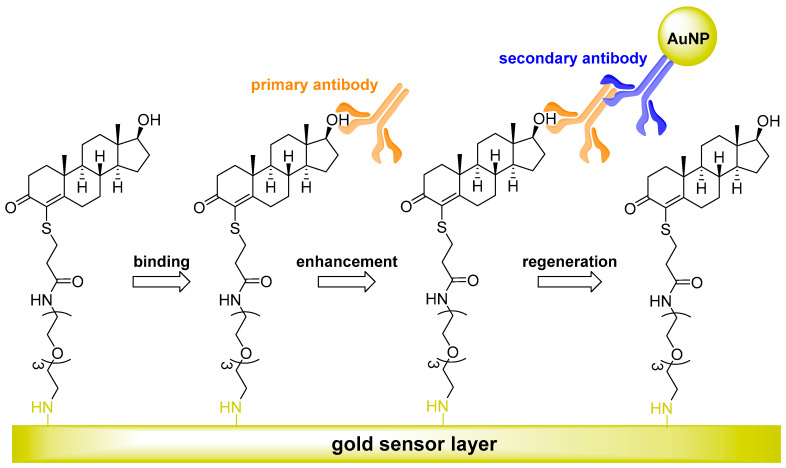
A diagram depicting the principle of a binding process in a surface plasmon resonance (SPR) immunosensor assay with nanogold labeling. An amino-terminated oligo(ethylene glycol)-linked testosterone conjugate was synthesized and immobilized on an SPR biosensor. The immunosensor system for testosterone utilized both secondary antibody and gold nanoparticle (AuNP) signal enhancement. The mechanism for the increased sensitivity resulted from increased binding mass and an Au–plasmon coupling effect. The addition of a secondary antibody with an attached AuNP increased the signal sensitivity of the assay by 12.5-fold compared to the primary antibody alone. The biosensor was stable for more than 330 binding and regeneration cycles [107].

**Figure 4:**
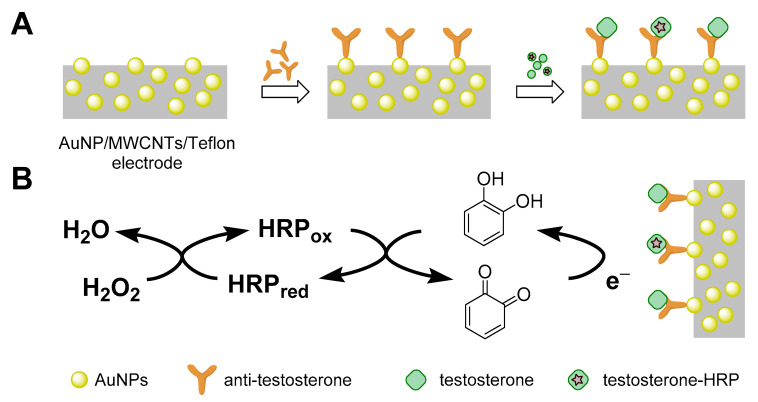
A diagram depicting the principle of an electrochemical testosterone immunosensor using AuNPs (gold nanoparticles)/multi-walled carbon nanotubes (MWCNTs)/Teflon electrodes. Anti-testosterone antibodies were directly attached to the hybrid electrode surface through the interaction of antibody thiol groups with AuNPs (**A**). A competitive assay between testosterone and testosterone conjugated to horseradish peroxidase (HRP–testosterone) was used for binding sites of antibodies. Amperometry at −0.05 V vs. Ag/AgCl was used to monitor affinity reactions upon the addition of H_2_O_2_ with catechol as a redox mediator (**B**) [104].

**Figure 5:**
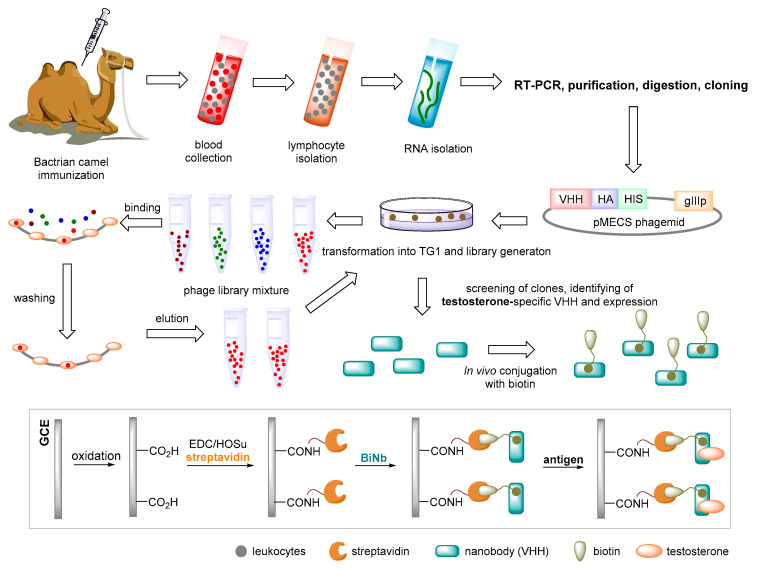
The anti-testosterone nanobody identification process. Bactrian camel immunization, VHH (nanobody, the antigen-binding fragment of heavy-chain-only antibodies), phage display library construction; biopanning, expression, and purification of soluble nanobodies (Nbs); a thermostability experiment; solvent effect; surface plasmon resonance affinity detection; biotinylation of a nanobody in vivo (BiNb), and development of a nanobody-based electrochemical immunosensor (i.e., immunogen or preparation, GCE: glassy carbon electrode, EDC: 1-ethyl-3-(3-dimethylaminopropyl)carbodiimide, HOSu: *N*-hydroxysuccinimide; cyclic voltammetry and electrochemical impedance spectroscopy measurements). RT-PCR: real-time polymerase chain reaction [101].

**Figure 6:**
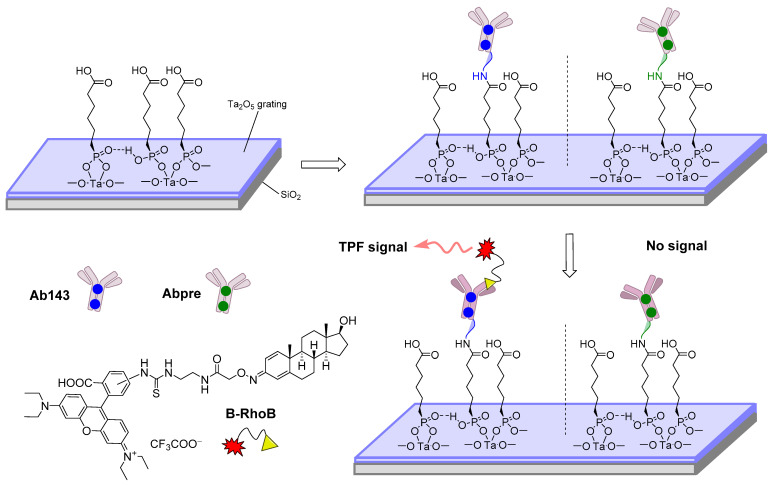
Diagram showing the surface functionalization, covalent immobilization of antibodies (Ab143: specific antibody marked with blue dots, Abpre: non-specific antibody marked with green dots), and the principle of a bioaffinity assay (TPF: two-photon fluorescence emission) using boldenone fluorescently labeled with rhodamine B (B-RhoB) [103].

The principle of an SPR immunosensor is schematically illustrated in Figure 3, while the principle of operation of an electrochemical immunosensor in Figure 4 and Figure 5 deals with the development of an immunosensor based on nanobodies. Furthermore, an immunosensor in which fluorescent antigen labeling is utilized is depicted in Figure 6. More detailed information on the general properties of electrochemical immunosensors of different arrangements and transduction strategies can be found, for example, in [112], as amperometric-type immunosensors based on screen-printed electrodes can be found in [113].

### 3.5. Androgen-Receptor- and Cell-Based Methods for AAS Determination

**Figure 7:**
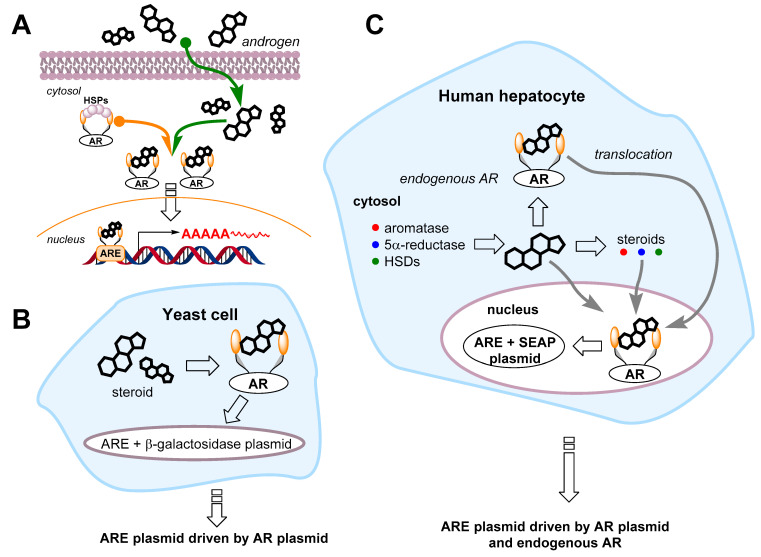
A diagram depicting the androgen response in cells. (**A**): androgens cross the cell plasma membrane to the cytosol and bind to the androgen receptor (AR). In the cytosol, the AR is held by heat shock proteins (HSPs) and other cofactors. Once androgens are bound to the AR, a conformational change is induced. The AR gets rid of inhibitory factors to form an androgen/AR complex. The complex translocates to the cell nucleus, and the receptor dimerizes and binds to the androgen response elements (AREs) located in the regulatory regions of target genes. When bound to the deoxyribonucleic acid (DNA), the AR enhances gene transcription by the ribonucleic polymerase. Yeast (**B**) and mammalian (**C**) cell-based androgen bioassays. The assays are based on the transfection of two plasmid DNAs: The first is the androgen receptor (AR) expression system providing AR expression in cells (yeasts do not express any endogenous ARs, and hepatocytes express them only at a minimal level). The second vector is the ARE-driven reporter gene vector. The most efficient reporter genes are β-galactosidase and secreted alkaline phosphatase (SEAP) in yeast and mammalian cells, respectively. Yeast cells do not express androgen-metabolizing enzymes, while human hepatocytes express a variety of them, including 5α-reductase, aromatase, and hydroxysteroid reductase (HSD) [115].

Another possibility for AAS determination lies in the fact that this group of substances achieve their anabolic effects by activating the androgen receptor (AR). The use of this phenomenon, with a proper methodology, offers the possibility of pan-androgenic determination, which is based not on the structure assessment, but on the effect induced. The use of ARs in cell-based bioassays has attracted the attention of several research groups [114,115]. Figure 7 describes the principle of utilizing yeast and mammalian cells for AR-based assays. Among others, Bailey et al. [63] developed an AR cell-based bioassay for monitoring androgenic activity; in this study, the androgenic glucuronidase activity of pretreated urine samples was measured using fluorescence emission of the AR expressed in fusion with the yellow fluorescent protein (YFP) and shown as testosterone equivalents. As expected, the AR was activated by all 17 evaluated AASs, but not the other steroids. Similarly, the AR activity was not induced by 12 metabolites of commonly abused AASs [63].

### 3.6. Oligonucleotide-Based Approaches for AAS Determination

By appropriate selection of a short, single-stranded oligonucleotide, it is possible to obtain a highly specific molecular recognition tool that can find application in the development of analytical methods. These molecules, also called aptamers, are often compared to antibodies for their high specificity. They are advantageous mainly because of their smaller sizes, lower cost, and stability at room temperature [116]. Regarding aptamers specific to AASs, a testosterone-binding aptamer was obtained and subsequently characterized using a modified systematic evolution of ligands via an exponential enrichment approach [117]. This methodology is thoroughly reviewed in [118]. Another aptamer, originally selected for 17β-estradiol, was used to develop a split aptamer-based sandwich fluorescence resonance energy transfer assay for 19-nortestosterone; although the aptamer used showed lower binding to 19-nortestosterone than to the originally intended molecule, the aptamer could be quantified by a suitable fluorophore or quencher to determine the analyte as a function of a decrease in fluorescence emission intensity by a method with an LOD of 5 µM [119].

**Figure 8:**
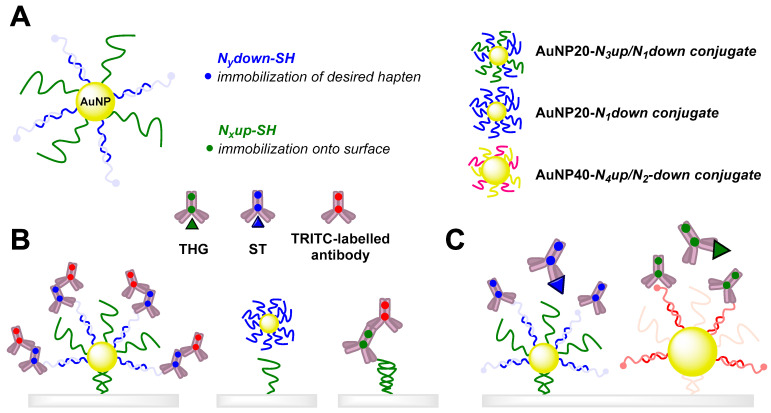
A diagram depicting the composition of multifunctional biohybrid nanoparticles. (**A**): A nanoparticle (NP) is codified with two different oligonucleotide strands: one for selective functionalization with the corresponding hapten, and the second for immobilization on a DNA microarray. The oligonucleotides are designated as *Nx* plus the words down or up. The “down” series hybridize with the corresponding hapten–oligonucleotide conjugate with the complementary oligonucleotide sequence. The “up” series hybridize with the complementary oligonucleotide sequence immobilized on the DNA microarray chip for site codification of the gold nanoparticles (AuNPs). AuNP20-*N*_3_up/*N*_1_down (20 nm-sized NPs) detect stanozolol (ST) with *N*_3_upSH, which is complementary to *N*_3_downNH_2_ oligonucleotides immobilized on the chip, and *N*_1_down, complementary to the hapten oligonucleotide probe 8-*N*_1_up. AuNP20-*N*_1_down does not have a chain that hybridizes with the DNA chip. AuNP40-*N*_4_up/*N*_2_down (40 nm-sized NPs) detect tetrahydrogestrinone (THG) and are biofunctionalized with *N*_4_upSH for hybridization with the *N*_4_downNH_2_ chains of the DNA chip, and with *N*_2_downSH for hybridization with the hapten oligonucleotide probe hG-*N*_2_up). Selectivity of the DNA-directed immobilization of AuNPs is demonstrated by fluorescence immunoassay and the multiplexed localized surface plasmon resonance microarray chip for the determination of ST and THG. (**B**): the diagram shows the experimental conditions for each case of the oligonucleotide-codified AuNPs, the antibodies used, and the multiplexed LSPR immunosensor chip. (**C**): specific antibodies bind to their corresponding hapten immobilized on the surface of the chip, or to a free analyte [122].

Advances in the determination of AASs using deoxyribonucleic acid (DNA) and Abs are well documented by Tort et al. [120,121], whose long-term development of a methodology for the competitive determination of stanozolol, tetrahydrogestrinone, and boldenone uses specific oligonucleotides to immobilize haptens on the surface of a microarray usable for an immunoassay. After binding of specific antibodies, quantification was performed using a fluorescently labeled secondary antibody [120]. A shift in the methodology for determining the same analytes has been the introduction of an SPR chip and associated detection with the similar use of specific DNA molecules to immobilize haptens [121]. So far, the latest update of the methodology from the same authors consists, among other things, of DNA-directed immobilization of multifunctional DNA–gold nanoparticles [122]. In Figure 8, there is a scheme of the method principle.

### 3.7. Enzyme-Based Sensor for AAS Determination

Another possible method to determine AASs is the development of a sensor using an enzyme. The developed sensor for amperometric determination of androsterone was based on the enzyme 3α-hydroxysteroid dehydrogenase, which was immobilized on the surface of a composite electrode formed by multi-walled carbon nanotubes, octylpyridinium hexafluorophosphate ionic liquid, and an oxidized form of nicotinamide adenine dinucleotide (NAD^+^) as a cofactor. The mentioned electrochemical detection was based on NADH produced during the enzymatic reaction. The linear working range of the method is 0.5–10 µM, with an LOD of 0.15 µM. This sensor gave satisfactory results when detecting androsterone in human serum [123].

### 3.8. Chemically Designed Artificial Sensors for AAS Determination

Chemically designed artificial sensors represent an exceptionally multidisciplinary approach for the determination of AASs. This is a modern approach using a variety of structures to specifically interact with the analyte of interest, following the pattern of antigen-antibody binding. As a result of the binding of the analyte to a suitable structure, a change will occur in the given system [124]. From the point of view of detection, the architecture of the given sensor is crucial, from which the nature of the monitored physical quantity is derived. Table 5 provides an overview of recently published chemically designed artificial sensors for the determination of AASs.

**Figure 9:**
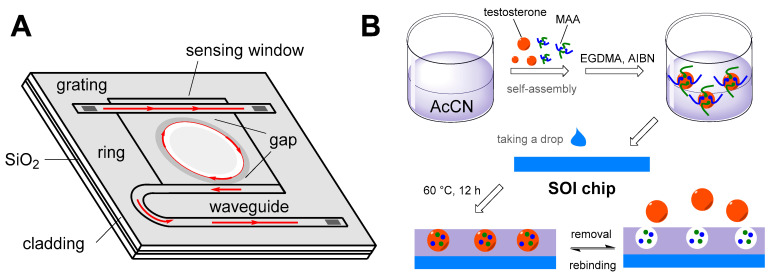
A diagram showing the description of individual parts of a micro-ring resonator sensor (**A**) and the principle of the preparation of molecularly imprinted polymers on the chip surface (**B**); AcCN: acetonitrile, MAA: methacrylic acid, EGDMA: ethylene glycol dimethacrylate, AIBN: 2,2′-azobis(2-methylpropionitrile), SOI: silicon-on-insulator wafer) [135].

**Figure 10:**
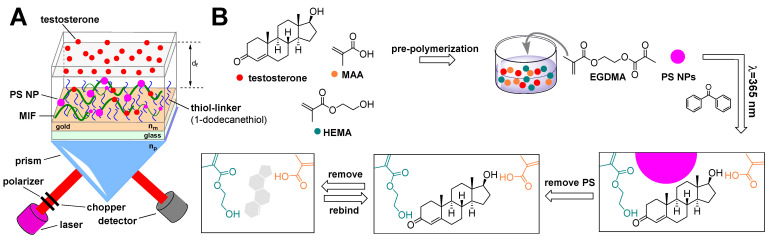
A diagram showing the setup of a surface plasmon resonance sensor (**A**), the PSNPs (polystyrene nanoparticles)–MIF (molecularly imprinted film)-functionalized sensor, and the schematic procedure of macroporous MIF formation (**B**). MIF was synthesized by photopolymerization of methacrylic acid (MAA), 2-hydroxyethyl methacrylate (HEMA), ethylene glycol dimethacrylate (EGDMA), and polystyrene nanoparticles (PSNPs) in combination with testosterone template molecules. This MIF-based sensor showed high stability and reproducibility for eight months when stored at room temperature [138].

Most of these artificial sensors are aimed at determining testosterone. In terms of their architecture, molecularly imprinted polymer (MIP)-based structures are a common recognition element. An example can be seen in Figure 9, in which this type of structure is prepared on the surface of the chip micro-ring resonator sensor, using the resonant wavelength shift for testosterone detection, with an LOD in the order of tens of picograms per mL. Another example demonstrating the variability of MIP utilization is shown in Figure 10; in this case, a macroporous MIP is used in combination with polystyrene nanoparticles on an SPR sensor, which is characterized by months-long stability at room temperature with a low LOD reaching femtograms per mL. In addition to the already mentioned transduction principles, the following approaches are also used for AAS determination: cyclic voltammetry, electrochemical impedance spectroscopy, differential pulse voltammetry, square-wave adsorptive stripping voltammetry, conductance, and localized SPR.

**Figure 11:**
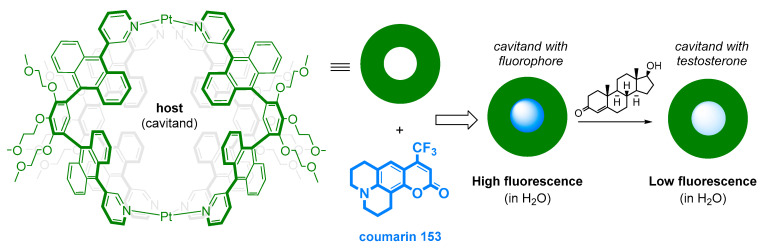
A diagram showing the molecular structure of the host (in green) and guests (coumarin 153 in blue, testosterone) used in the nanogram-scale fluorescent detection of testosterone. The fluorescent cavitand had the emission at λ = 423 nm (using λex = 356 nm) [128].

**Figure 12:**
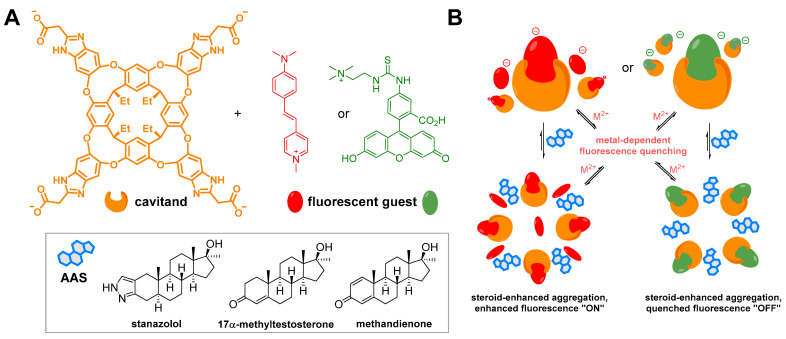
A diagram showing the molecular structure of the host (in orange), fluorescent guests (in red, *trans*-4-[4-(dimethylamino)styryl]-1-methylpyridinium iodide (DSMI), and a fluorescein-based dye in green), and tested anabolic-androgenic steroids (AASs; (**A**)). Possible aggregation modes of the complexes and the effects of steroid addition on the emission profiles (**B**). The sensing is triggered by an aggregation mechanism. Aggregation can be mediated by the presence of both metal ions and steroids. Both the “turn-on” and “turn-off” modes of fluorophores are essential for analyte discrimination [127].

Another approach to AAS determination based on a chemically designed artificial sensor is shown in Figure 11. This methodology is based on the host structure and fluorescent guests, which enable nanogram-scale fluorescent detection of testosterone. Figure 12 shows the similar principle of the host structure and fluorescent guests that mediate fluorescent quenching depending on the presence of metal ions or selected steroids. This highly selective method achieves sensitivity in the order of 10 µM.

## 4. Conclusions

This article deals with the procedure for determining AASs, which represent a socially highly problematic and risky group of biologically active substances. Given the fundamental importance of testosterone for the human body, and the fact that other AASs are derived from it, it is not surprising that a large number of recently published methodologies for AAS determination focus on this hormone. Methods for the detection of testosterone’s most abused derivatives—such as nandrolone, stanozolol, boldenone, and several others—are not neglected.

In addition to the principal importance of chromatographic methods for AAS determination, Ab-based methods are also widely used. Combining these traditionally used approaches, such as by concentrating samples with immunoaffinity sorbents before chromatographic analysis, might also be beneficial. An already confirmed trend in the development of Ab methods for the determination of AASs is the departure from radioactive labeling, which to some extent has replaced enzyme labeling. Most recently developed EIAs are in the ELISA format, and the popularity of this methodology for AAS determination is reflected in both the number of reported methods and the portfolio of their analytes of interest. The most user-friendly method for AAS determination in general, although of a semi-quantitative nature, is LFIA, which can be used in fieldwork for its time efficiency and equipment simplicity, since a naked eye is sufficient for its evaluation.

Efforts to increase the analytical performance of traditional Ab methods have resulted in the development of novel multidisciplinary methods for mediating the interaction of antibodies with the analyte of interest to obtain a detectable signal, and it is the numerous treatments of immunosensors that use a variety of materials to immobilize the immunoreagent that provide results faster, with higher reproducibility, and with smaller sample volumes compared to conventional ELISAs. The sensitivity of these methods—which, in addition to the architecture of the sensor itself and the signal transduction system, depends significantly on the Abs used—is of a similar order as that achieved by ELISA.

State-of-the-art immunosensor development techniques utilize the selectivity of not only antibodies but also oligonucleotides, which can specifically bind to a target molecule. By simultaneous utilization of gold nanoparticles, this approach has brought self-organizing chips designed for the robust and selective determination of different AASs at the same time.

Attractive results are obtained by ARs using methods that are promising in terms of much-needed non-target detection. Such methods are based not on the recognition of the structure, but the effect of the substance. Therefore, this approach might be beneficial for the development of group-specific methods.

Efforts towards single-molecule-specific AAS binding have resulted in the development of chemically designed artificial structures used as sensors. The so-called molecularly imprinted polymers and their films, which recognize AASs with high specificity, are broadly utilized. They are often used in combination with similar materials, and in arrangements known to immunosensors using a wide portfolio of transduction principles. In extreme cases, these sensors can reach down to (sub)femtomolar detection limits.

Another modern approach in the determination of AASs uses chemically generated host structures of macromolecular character, which can non-covalently interact with the analyte of interest via hydrogen bonds, van der Waals forces, and hydrophobic interactions in the internal cavity of the host structure. Such a procedure increases the solubility of lipophilic AASs in aqueous media, which is essential for the possibility of direct analysis of biological fluids. A critical point in the determination of AASs then brings the use of such structures for the host-guest displacement assay, in which the target analyte “pushes” the fluorophore out of the host structure under detectable fluorescence modulation within a single molecule.

In conclusion, the requirements for forensic, biomedical, environmental, food, and beverage AAS analyses have evolved very rapidly. In overcoming the complicated analytical challenges related to the need for a fast, simple, inexpensive, portable, and highly specific method for AAS determination in matrices of various origins, professional efforts are certainly moving in the right direction. However, despite this relentless effort and brilliant advancements in technological approaches to the determination of AASs, we do not have yet an absolutely convenient method.

## Figures and Tables

**Table 1:** Immunoaffinity columns for the determination of anabolic-androgenic steroids.

Compound of Interest	Approach and Ab Used	Analytical Characteristics	Matrix	Ref.
Methandienone	Monoclonal Ab covalently bound to chitosan by a glutaraldehyde linker	MBC of an adsorbent was 3900 ng·mL^−1^	Spiked animal tissue and feed samples	[80]
Methandienone	Monoclonal Ab against methandienone-KLH coupled to CNBr-activated Sepharose 4B (commercially available)	MBC of an adsorbent was 4760 ng·mL^−1^	Spiked animal tissue and feed samples	[81]
Methandienone	Polyclonal	MBC of an adsorbent was 334 ng·mL^−1^	Spiked animal tissue and feed samples	[82]
Epitestosterone	Half-IgG of anti-epitestosterone monoclonal antibodies were covalently immobilized onto Fe_3_O_4_ magnetic nanoparticles coated with gold	Pretreatment of urine samples by this novel immunoaffinity column led to an increase in the sensitivity of HPLC analysis by two orders of magnitude (LOD = 60 pg·mL^−1^)	Human urine	[83]

Ab: antibody; HPLC: high-performance liquid chromatography; IgG: immunoglobulin G; KLH: keyhole limpet hemocyanin; MBC: maximum binding capacity.

**Table 2:** Enzymatic immunoassays for the determination of anabolic-androgenic steroids.

A Compound of Interest/EIA Format	Immunogen/Coating Antigen	Antibodies	Analytical Characteristics of the Most Sensitive System	Specificity of the Most Sensitive System/Determined Cross-Reactants > 1%	Matrix	Ref.
Stanozolol/ ELISA	Three different BSA-derived immunogens/ biotinylated, BSA- and RSA-derived antigens	Eight batches of rabbit polyclonal Ab	IC_50RSA_ = 0.32 ng·mL^−1^ LOD_RSA_ = 20 pg·mL^−1^ LWR_RSA_ = 0.03–3.53 ng·mL^−1^ IC_50Biotin_ = 3.9 ng·mL^−1^ LOD_Biotin_ = 570 pg·mL^−1^ LWR_Biotin_ = 1.1–24.5 ng·mL^−1^	Group-specific to 17α-methylated AAS	Dietary supplements	[40]
Methyltestosterone/ ELISA	BSA-derived immunogen/ OVA-derived antigen	Eight murine polyclonal/ one monoclonal Ab	IC_50_ = 0.3–4.4 µg·L^−1^ LOD = 37.2–697.8 ng·L^−1^ LOQ = 70.0–1524.0 ng·L^−1^	Nortestosterone, testosterone, and trenbolone	11 types of animal tissues	[87]
DHEA/ ELISA		Rabbit polyclonal Ab	IC_50_ = 4.89 ng·mL^−1^ LOD = 0.1 ng·mL^−1^ LWR = 0.41–58.77 ng·mL^−1^	Androstenedione	Slimming products (teas, capsules, tablets)	[88]
Mesterolone/ ELISA	BSA-derived immunogen and antigen		IC_50_ = 4.2 ng·mL^−1^ LOD = 10 pg·mL^−1^ LWR = 1–34 ng·mL^−1^	Dihydrotestosterone, testosterone, progesterone, boldenone sulfate, 4-androstene-3,17-dione, nandrolone, methandienone, boldenone undecanoate, epitestosterone, oxandrolone, trenbolone, dehydroepiandrosterone	Dietary supplements	[89]
Methandienone/ELISA	BSA-derived immunogen/ OVA-derived antigen		IC_50_ = 1.54 ng·mL^−1^ LOD = 40 pg·mL^−1^ LWR = 0.2–12 ng·mL^−1^	Boldenone and its derivatives, testosterone and its derivatives, 4-androstene-19-ol-3,17-dione, cortisone, 4-androsten-3,17-dione, 11-deoxycorticosterone		[38]
Nandrolone and testosterone/ ELISA	Four BSA-derived immunogens/ linker-optimized biotinylated nandrolone and testosterone as antigens	Four batches of rabbit polyclonal Ab	The most sensitive nandrolone-based system: IC_50_ = 180 pg·mL^−1^ LOD = 4 pg·mL^−1^ LWR = 0.02–1.38 ng·mL^−1^	CR in respect to nandrolone: testosterone, dihydrotestosterone, drostanolone, trenbolone, boldenone		[39]
Boldenone/ ELISA	BSA-derived immunogen/ OVA-derived antigen	Rabbit polyclonal Ab	IC_50_ = 293 pg·mL^−1^ LOD = 14 pg·mL^−1^ LWR = 0.065–1.52 ng·mL^−1^	Boldenone and its derivatives, dihydrotestosterone, methandienone, testosterone		[37]
Stanozolol/ CLEIA using luminol		Two batches of rabbit polyclonal Ab	IC_50_ = 340 pg·mL^−1^ LOD = 70 pg·mL^−1^	Oxymetholone, testosterone	Various plant and animal tissues	[90]
Methyltestosterone/ELISA		Murine monoclonal Ab	IC_50_ = 260 pg·mL^−1^ LOD = 45 pg·mL^−1^ LWR = 0.02–1.38 ng·mL^−1^	Testosterone, nortestosterone	Animal feed	[91]
Methandienone/ELISA	BSA-derived immunogen/KLH-derived immunogen	Murine monoclonal Ab	IC_50_ = 7.89 ng·mL^−1^ LOD = 0.17 ng·mL^−1^	n.a.	n.a.	[81]
Stanozolol, boldenone and tetrahydrogestrinone/ELISA	Multianalyte ELISA/four BSA-derived immunogens/three BSA-derived antigens	Cocktail of three rabbit polyclonal Abs	IC_50_ = 0.16–9.75 ng·mL^−1^ LOD = 20–340 ng·mL^−1^	Detection of up to 11 AASs	Human serum	[92]
Nandrolone/ ELISA	BSA-derived immunogen/OVA-derived antigen	Murine monoclonal Ab	IC_50_ = 0.52 ng·mL^−1^ LOD = 0.01 ng·mL^−1^ LWR = 0.03–38 ng·mL^−1^	17α-Nortestosterone, trenbolone, β-boldenone	Beef and pork tissues	[93]
Stanozolol, boldenone, methylboldeno-ne, tetrahydrogestrinone/ELISA	Multiple ELISA (combination of 8 assays)/ 8 BSA-derived antigens/multiple component analyses calculation	Six rabbit polyclonal Abs	IC_50_ = 0.38–2.60 nM LOD = 0.1–316 nM	Detection of up to 23 AASs	Human serum and urine	[94]
Stanozolol, 6β-hydroxy-stanozolol/ ELISA	Immunosorbent solid phase as a pre-step/BSA- derived immunogen/ coated with antiserum	Two rabbit polyclonal Abs	Values for stanozolol: IC_50_ = 550 ng·mL^−1^ LOD = 36 ng·mL^−1^ LWR = 104–2720 ng·mL^−1^	CR in respect to stanozolol: 16β-hydroxystanozolol, norstanozolol, 3′-hydroxystanazolol, boldenone, methylboldenone	Cow urine	[84]

BSA: bovine serum albumin; CLEIA: chemiluminescence enzyme immunoassay; CR: cross-reactivity; DHEA: dehydroepiandrosterone; EIA: enzyme immunoassay; ELISA: enzyme-linked immunosorbent assay; IC_50_: half-maximal inhibitory concentration; LOD: limit of detection; LOQ: limit of quantification; LWR: linear working range; KLH: keyhole limpet hemocyanin; n.a.: information not available; OVA: ovalbumin; RSA: rabbit serum albumin.

**Table 3:** Lateral flow immunoassays (LFIAs) for the determination of anabolic-androgenic steroids.

Compound of Interest	Approach and Used Ab	Analytical Characteristics	Matrix	Ref.
17α-Methylated AASs	Gold-labeled rabbit polyclonal	LOD = 0.7 ng·mL^−1^	Dietary supplements	[41]
Dehydroepiandrosterone		LOD = 500 µg·kg^−1^	Slimming products (herbal teas, capsules, pills)	[88]
Mesterolone		LOD = 50 ng·mL^−1^	Dietary supplements	[89]
Methyltestosterone	Gold-labeled murine monoclonal	LOD = 1 ng·mL^−1^	Animal feed	[91]
Nandrolone	Gold-labeled rabbit polyclonal	LOD = 1 ng·mL^−1^	Dietary supplements	[39]
Nandrolone	Gold-labeled murine monoclonal	LOD = 1 ng·mL^−1^	Beef and pork tissues	[93]

LOD: limit of detection.

**Table 4:** Immunosensors for the determination of anabolic-androgenic steroids.

Compound of Interest	Type of Transduction and Its Principle	Description of Methods and Materials Used	Analytical Characteristics	Matrix	Ref.
Testosterone, DHEA	Electrochemical/ amperometric	Anti-testosterone Abs/glutaraldehyde/the polymer drop-coated screen-printed carbon electrode surface	LOD = 16.7 ng·mL^−1^ LWR = 10–500 ng·mL^−1^	Synthetic urine and synthetic serum	[95]
Testosterone	Electrochemical/ impedance spectroscopy	Anti-testosterone Abs/Au(3-mercaptopropionic acid)/ (3-aminopropyl) triethoxysilane/indium tin oxide glass electrode	LOD = 3.9 ng·mL^−1^ LWR = 10–500 ng·mL^−1^	Saliva	[100]
Testosterone	Electrochemical/ impedance spectroscopy	Isolation of Bactrian nanobody from an immune phage display library/ biotinylation/glassy carbon electrode	LOD = 0.045 ng·mL^−1^ LWR = 0.05–5 ng·mL^−1^	Serum	[101]
Testosterone	Electrochemical/ amperometric	Screen-printed carbon electrodes and protein-A-functionalized magnetic beads/testosterone labeled with HRP/ hydroquinone as the redox mediator	LOD = 1.7 pg·mL^−1^ LWR = 0.005–50 ng·mL^−1^ EC_50_ = 250 pg·mL^−1^	Human serum	[102]
Methylboldenone	Optical/ two-photon fluorescence emission	Immunoreagents/immobilized onto a resonant Ta_2_O_5_ double -grating waveguide structure	LOD = 0.1 ng·mL^−1^ IC_50_ = 4.6 ng·mL^−1^	Buffer	[103]
Testosterone	Electrochemical/ amperometric	Testosterone and HRP-testosterone/Abs on AuNPs/MWCNTs/Teflon electrodes/H_2_O_2_ with catechol as redox mediator	LOD = 85 pg·mL^−1^ LWR = 0.1–10 ng·mL^−1^	Human serum	[104]
Testosterone	Electrochemical/ chronoamperometric	3D competitive sensing platforms/gold disc-ring microelectrode array for immunofunctionalization/near second microelectrode array for electrochemical monitoring	LOD = 12.5 pg·mL^−1^ LWR = 0.01–10 ng·mL^−1^	Human saliva	[105]
Stanozolol and methylboldenone	Electrochemical/ amperometric, voltammetric	Two specific Abs/arrays of carbon nanotube field-effect transistors	Only recognition	Optimal conditions	[106]
Testosterone	Optical/ surface plasmon resonance	Testosterone/oligoethylene glycol/ surface plasmon resonance biosensor/secondary Abs and AuNP signal enhancement	LOD = 15.4 pg·mL^−1^ LWR = 29–290 pg·mL^−1^	Human saliva	[107]
Testosterone	Electrochemical/ potentiometric	Anti-testosterone Abs/polyvinyl butyral sol–gel film doped with gold nanowires	LOD = 0.1 ng·mL^−1^ LWR = 1.2–83.5 ng·mL^−1^	Human serum	[108]
Stanozolol	Electrochemical/ chronoamperometric	Immobilized antigen–protein conjugate on screen-printed electrodes	LOD = 41.6 pg·mL^−1^ LWR = 0.2–500 ng·mL^−1^ EC_50_ = 2.15 ng·mL^−1^	Bovine urine	[109]
Nandrolone and methyltestosterone			19-Nortestosterone: LOD = 10.5 pg·mL^−1^ EC_50_ = 936 pg·mL^−1^ methyltestosterone: LOD = 14.8 pg·mL^−1^ EC_50_ = 274 pg·mL^−1^		[110]
Testosterone		Immobilized testosterone conjugate on screen-printed electrodes/ anti-testosterone Abs fragments	LOD = 90 pg·mL^−1^ LWR = 0.3–40 ng·mL^−1^		[111]

Abs: antibodies; DHEA: dehydroepiandrosterone; EC_50_: half-maximal effective concentration; HRP: horseradish peroxidase; IC_50_: half-maximal inhibitory concentration; LOD: limit of detection; LOQ: limit of quantification; LWR: linear working range; MWCNTs: multiwalled carbon nanotubes; AuNPs: gold nanoparticles; SPEs: screen-printed electrodes; SPCEs: screen-printed carbon electrodes.

**Table 5:** Chemically designed artificial sensors for the determination of anabolic-androgenic steroids.

Compound of Interest	Principle of Transduction or Detection	Description of Method and Used Materials	Analytical Characteristics	Matrix	Ref.
Testosterone	Cyclic voltammetry	Synthetic self-assembly of poly(aniline-co-metanilic acid) and testosterone forming imprinted electronically conductive polymers on sensing electrodes	LOD = units of pM LWR = 0.1–100 pg·mL^−1^	Urine	[125]
Mesterolone, oxandrolone, oxymetholone, stanozolol, trenbolone	Fluorescence modulation	β-Cyclodextrin-promoted interactions between the analyte of interest and fluorescent rhodamine 6G, leading to analyte-specific changes in the fluorophore emission signal	LOD = 0.775–17 µM specificity = 100% differentiation between structurally similar analytes	Citrate buffer	[126]
Stanozolol, 17α-methyltestosterone, methandienone		Arrayed complexes of host-guest cavitands using two fluorescent indicators and a low amount of small metal ions	LOD = 10 µM; highly selective, able to discriminate between structures varying only by a single π bond	Human urine	[127]
Testosterone	Fluorescent detection	Fluorescent detection of testosterone by a receptor-dye complex. The emission of a fluorescent coumarin derivative as a dye guest is displaced by a more hydrophobic hormone guest	Discrimination between testosterone and female hormones in the order of molecule units	Water	[128]
	Electrochemical impedance spectroscopy	Microstructures of molecularly imprinted polymers on functionalized nanocrystalline diamond/ testosterone target molecule/ *N,O*-bismethacryloyl ethanolamine as a bifunctional monomer	LOD = 0.5 nM LWR = 0.5–20 nM	Human urine and saliva	[129]
	A photoinduced electron transfer fluorescent probe system	Covalently linking β-cyclodextrin to the surface of N, S co-doped carbon dots/carbon dot and (ferrocenylmethyl)trimethylammonium iodide (Fc^+^)	LOD = 0.51 μM LWR = 0–280 μM	Water and cytoplasm	[130]
Testosterone	Electrochemical impedance spectroscopy	Nanosized molecularly imprinted polymer film that was electrochemically grafted on a graphene oxide sheet/modified glassy carbon electrode	LOD = 0.4 fM LWR = 1 fM–1 µm	Human serum	[131]
	Differential pulse voltammetry	Electrochemical reduction of testosterone in the presence of a cationic surfactant using graphene oxide/glassy carbon electrode	LOD = 0.1 nM LWR = 2–210 nM	Human plasma and urine	[132]
Testosterone, nandrolone, nandrolone-17- propionate	Fluorescence emission-based binding assays	Cucurbit[*n*]urils as a high-binding -capacity host provide water-soluble formulations for an analyte of interest. Displacement of a fluorescent dye by various steroidal analytes provides a distinct and measurable fluorescent response	LOD = units of µM	Water, buffer, gastric acid, blood serum	[133]
Testosterone	Square-wave adsorptive stripping voltammetry	Bismuth film/ glassy carbon electrode	LWR = 1–45 nmol·L^−1^ LOD = 0.3 nmol·L^−1^ and 0.09 ng·mL^−1^	Oil-based pharmaceuticals and human urine	[134]
Testosterone	Resonant wavelength shift	Micro-ring resonator sensor with MIP	LWR = 0.05–10 ng·mL^−1^ LOD = 48.7 pg·mL^−1^	Deionized water	[135]
Testosterone	Surface plasmon resonance	Double photografting polymerization of 1-dodecanethiol leading to a double layer of MIF on the gold surface of SPR sensor chips	LWR = 1 × 10^−12^–1 × 10^−8^ mol·L^−1^ LOD = 10^−12^ mol·L^−1^	Seawater	[48]
	Square-wave adsorptive stripping voltammetry	Glassy carbon electrode in the presence of cationic surfactant	LWR = 10–70 nM LOD = 1.2 nM	Oil-based pharmaceuticals and human urine	[136]
	Cyclic voltammetry	Oxidation of testosterone at the plane glassy carbon electrode modified with cobalt oxide	LWR = 0.33 to 2.00 µM LOD = 0.16 µM	Supporting electrolyte (0.10 M NaOH)	[137]
Testosterone	Surface plasmon resonance	Gold-chip-based macroporous molecularly imprinted film in combination with polystyrene nanoparticles	LOD = units of fg·mL^−1^	Artificial urine and human urine	[138]
Testosterone	Electrochemical impedance spectroscopy	MIP was synthetized at the surface of gold electrodes via a photoradical initiator covalently coupled with a self-assembled monolayer of amine-terminated alkanethiol	Linearity up to 50 µg·L^−1^ LOD = 103 ng·L ^−1^	PBS buffer	[139]
Testosterone, epitestosterone	Square-wave voltammetry	Bare and single-wall carbon nanotubes modified an edge plane of a pyrolytic graphite electrode	LOD_T_ = 2.8 × 10^−9^ M LOD_ET_ = 4.1 × 10^−9^ M LWR_T&ET_ = 5–1000 nM	Human urine	[140]
Nandrolone		Fullerene modified an edge plane of a pyrolytic graphite electrode	LWR = 0.01–50 nM LOD = 1.5 × 10^−11^ M	Medicinal samples	[141]
19-Norandrostendione	Conductance	Chemically modified Δ^5^-3-ketosteroid isomerase immobilized on the surface of a silicon nanowire	LOD = units of fM	n.a.	[142]
Stanozolol	Localized SPR	Functionalized glass substrates by noble metal gold colloid	LOD = 0.7 μg·L^−1^ Dt = 2 min	Buffer solution	[143]

Dt: detection time; LOD: limit of detection; LWR: linear working range; MIF: molecularly imprinted polymer film; MIP: molecularly imprinted polymer; n.a.: not available; PBS: phosphate-buffered saline; SPR: surface plasmon resonance.

## Data Availability

Not applicable.

## Abbreviations

AASs	Anabolic-androgenic steroids
AcCN	Acetonitrile
AIBN	2,2’-Azobis(2-methylpropionitrile)
Ab	Antibody
AR	Androgen receptor
AREs	Androgen response elements
AuNP	Gold nanoparticle
BiNb	Biotinylation of a nanobody in vivo
BSA	Bovine serum albumin
CLEIA	Chemiluminescent enzyme immunoassay
CR	Cross-reactivity
DHEA	Dehydroepiandrosterone
DS	Dietary supplement
DSMI	*trans*-4-[4-(Dimethylamino)styryl]-1-methylpyridinium iodide
EC_50_	Half-maximal effective concentration
EDC	1-Ethyl-3-(3-dimethyl aminopropyl)carbodiimide
EGDMA	Ethylene glycol dimethacrylate
EIA	Enzyme immunoassay
ELISA	Enzyme-linked immunosorbent assay
GCE	Glassy carbon electrode
HEMA	2-Hydroxyethyl methacrylate
HPLC	High-performance liquid chromatography
HOSu	*N*-Hydroxysuccinimide
HRP	Horseradish peroxidase enzymes
HSD	Hydroxysteroid reductase
HSPs	Heat shock proteins
IAC	Immunoaffinity chromatography
IC_50_	Half-maximal inhibitory concentration
IgG	Immunoglobulin G
KLH	Keyhole limpet hemocyanin
LFIA	Lateral flow immunoassay
LOD	Limit of detection
LOQ	Limit of quantification
LWR	Linear working range
MAA	Methacrylic acid
mAb	Mouse-derived antibody
MIF	Molecularly imprinted polymer film
MIP	Molecularly imprinted polymer
MT	Methyltestosterone
MT-CMO-OVA	A conjugate of methyltestosterone-3-carboxymethyloxime with ovalbumin
MWCNTs	Multiwalled carbon nanotubes
NAD	Nicotinamide adenine dinucleotide
Nb	Nanobody
NC	Nitrocellulose
OVA	Ovalbumin
PSNPs	Polystyrene nanoparticles
RSA	Rabbit serum albumin
SEAP	Secreted alkaline phosphatase
SOI	Silicon-on-insulator wafer
SPCEs	Screen-printed carbon electrodes
SPEs	Screen-printed electrodes
SPR	Surface plasmon resonance
ST	Stanozolol
THG	Tetrahydrogestrinone
TLC	Thin-layer chromatography
TPF	Two-photon fluorescence
UOC	Under optimal conditions
VHH	The antigen-binding fragment of heavy-chain-only antibodies
WADA	World Anti-Doping Agency
